# Bibliographical Notices

**Published:** 1844-12-28

**Authors:** 


### BIBLIOGRAPHICAL NOTICES.

*An Introductory Lecture to the course of Institutes of Medicine, &c., in Jefferson Medical College, delivered November* 4, 1844. By Professor Dunglison. Published by the Class.

*Introductory for* 1844–'5, *On the present position of some of the most important of the Modern Operations of Surgery.* By Thomas D. Mütter, M. D. Published by the Class.

*Introductory Lecture to the Course of Chemistry, delivered in Jefferson Medical College, November* 6, 1844. By Franklin Bache, M. D. Published by the Class.

Of the value of these publications, for obvious reasons, we shall say but little. To others, whose judgment will not be suspected of partiality, we leave the privilege of speaking of their merits.

In the Introductory of Dr. Dunglison, after a warm-hearted salutation to the members of his class, some remarks on the facilities afforded for medical instruction by the city of Philadelphia, and the present condition of the School in which he is a professor, the author notices some of the modern doctrines in medicine, and closes with a few pertinent observations on the proper method of studying the science of medicine. The lecture is written in the easy flowing style peculiar to the author.

The title of Dr. Mütter's address sufficiently explains its character. The author, it is known, visited Europe during the past summer, and mingled much with the prominent surgeons on the other side of the Atlantic, and in this lecture he gives the opinions now entertained by them as to the merits of some of the late operations, of whose value the profession are yet in doubt.

Dr. Bache's lecture is what it has always seemed to us such a lecture *ought* to be—introductory to his course. The student is ushered at once into the laboratory, and takes a bird's eye view of the whole subject before him. Nothing is concealed. His task, herculean as it is, is at once laid before him, and he is invited to enter upon it with the assurance of success, through his own exertions and the ever willing assistance of his instructor.

